# Reconstructing individual-level exposures in cohort analyses of environmental risks: an example with the UK Biobank

**DOI:** 10.1038/s41370-023-00635-w

**Published:** 2024-01-08

**Authors:** Jacopo Vanoli, Malcolm N. Mistry, Arturo De La Cruz Libardi, Pierre Masselot, Rochelle Schneider, Chris Fook Sheng Ng, Lina Madaniyazi, Antonio Gasparrini

**Affiliations:** 1 School of Tropical Medicine and Global Health, Nagasaki University, Nagasaki, Japan; 2 Environment & Health Modelling (EHM) Lab, Department of Public Health Environments and Society, London School of Hygiene & Tropical Medicine, London, UK; 3 Department of Economics, Ca’ Foscari University of Venice, Venice, Italy; 4 Φ-lab, European Space Agency, Frascati, Italy; 5 Department of Global Health Policy, Graduate School of Medicine, The University of Tokyo, Tokyo, Japan

**Keywords:** Epidemiology, Exposure Modeling, Air pollution, Exposure linkage

## Abstract

**Abstract:**

Recent developments in linkage procedures and exposure modelling offer great prospects for cohort analyses on the health risks of environmental factors. However, assigning individual-level exposures to large population-based cohorts poses methodological and practical problems. In this contribution, we illustrate a linkage framework to reconstruct environmental exposures for individual-level epidemiological analyses, discussing methodological and practical issues such as residential mobility and privacy concerns. The framework outlined here requires the availability of individual residential histories with related time periods, as well as high-resolution spatio-temporal maps of environmental exposures. The linkage process is carried out in three steps: (1) spatial alignment of the exposure maps and residential locations to extract address-specific exposure series; (2) reconstruction of individual-level exposure histories accounting for residential changes during the follow-up; (3) flexible definition of exposure summaries consistent with alternative research questions and epidemiological designs. The procedure is exemplified by the linkage and processing of daily averages of air pollution for the UK Biobank cohort using gridded spatio-temporal maps across Great Britain. This results in the extraction of exposure summaries suitable for epidemiological analyses of both short and long-term risk associations and, in general, for the investigation of temporal dependencies. The linkage framework presented here is generally applicable to multiple environmental stressors and can be extended beyond the reconstruction of residential exposures.

**Impact:**

This contribution describes a linkage framework to assign individual-level environmental exposures to population-based cohorts using high-resolution spatio-temporal exposure. The framework can be used to address current limitations of exposure assessment for the analysis of health risks associated with environmental stressors. The linkage of detailed exposure information at the individual level offers the opportunity to define flexible exposure summaries tailored to specific study designs and research questions. The application of the framework is exemplified by the linkage of fine particulate matter (PM_2.5_) exposures to the UK Biobank cohort.

## Introduction

The role of environmental factors as determinants of health has gained importance in the last decades. Early epidemiological studies have investigated the health impacts of environmental stressors, in particular assessing the mortality risks associated with exposure to air pollutants such as particulate matter [1]. The evidence has been subsequently strengthened and extended to a variety of other exposures and outcomes [2, 3]. Emergent research also suggests health risks associated with other environmental exposures, such as other pollutants such as nitrogen oxides, temperature, pollen, and other chemicals [2, 4], as well as for a variety of health outcomes, including communicable and non-communicable disease [5].

A known problem in this research area is that most environmental stressors, while affecting entire populations and generating considerable health burdens, are usually associated with relatively low health risks at the individual level. Estimating such associations therefore requires large epidemiological studies. With few exceptions [6], early investigations relied on administrative databases with limited individual information and were often based on ecological designs [7]. Nowadays, new opportunities are offered by the availability of large population-based cohorts that match the recruitment of a high number of participants with the detailed reconstruction of individual information through linkage across multiple databases. Recent endeavours, such as the European EPIC study, the UK Biobank [8], and the Japanese JECS include the collection of detailed questionnaires and physical measurements, through which it is possible to explore small variations in susceptibility due to lifestyles, genetic traits, and other individual and contextual characteristics.

A related problem is represented by the exposure assessment. Direct personal monitoring of environmental exposures is unfeasible for large-scale studies across long periods of time, and therefore outdoor levels at residential locations are typically used as a proxy for personal exposure. Early cohort studies made use of data from sparse monitoring stations, which can result in misclassification and reduced exposure contrasts [1, 9], more so for exposure that features high spatial and/or temporal variability such as air pollution. Nowadays, exposure modelling techniques offer valuable solutions with improved prediction accuracy and coverage. For instance, modern methodologies can combine multi-domain predictors in sophisticated analytical models to derive high-resolution spatio-temporal maps over large regions [10]. These methods have been previously used to harmonise the exposure assignment to large population-based cohorts in North America [11] and Europe [12].

Such models nonetheless do not always produce temporally disaggregated measures [13], required for assessing short-term risks. Other studies have assigned annual exposure averages, but without accounting for residential changes and potential long-lagged associations with past exposures [6]. More informative and accurate exposure summaries can be defined by reconstructing the complete exposure history for each cohort participant. This extension offers the possibility to examine other aspects such as multiple association timescales and windows of susceptibility. However, this extension presents important methodological, logistical, and practical issues.

In this contribution, we present a currently applied framework for the linkage of highly resolved outdoor environmental exposures to large cohorts using individual residential information. The illustration provides the opportunity to discuss methodological aspects and technical requirements, as well as specific problems such as privacy constraints. We exemplify this process by assigning exposures to air pollution to the UK Biobank cohort, a large prospective study involving more than half a million participants. The article outlines a number of steps needed to generate individual-level exposure profiles, and finally to derive exposure summaries consistent with alternative study designs and research questions.

## Materials and methods

### UK Biobank

The UK Biobank cohort is a longitudinal study that has involved adults aged 40–69 at recruitment in the United Kingdom between 2006 and 2010 [8]. Overall, 503,325 participants were recruited and each of them attended an assessment centre and completed questionnaires on their socio-economic aspects, lifestyle factors, and medical history, among other information. They also underwent a wide range of physical measures, as well as the collection of biological samples. The study is periodically enriched with follow-up assessments, new sources of data originating from research projects, and updates from external databases. These comprise the linkage with electronic health records (EHR) and national health system registers, including death and cancer occurrences, hospitalisations and primary care visits. Information on environmental exposures currently available in the UK Biobank is represented by annual averages of air pollutants and noise for single years between 2006 and 2010. Air pollution measures are limited to a sub-group of participants and obtained from Europe-wide land-use regression models [14].

The linkage of new environmental data to cohort participants necessitates three sources of information, exemplified by the pseudo-data illustrated in Table 1. These simulated data are used in this and the next sections to describe the linkage process and epidemiological analyses. The first piece of information is about the baseline cohort information, illustrated in Table 1a. These data are represented here by the enrolment and last follow-up dates for each participant, identified by a pseudo-code. This usually is linked to other information collected at the baseline or during follow-up assessments, such as personal characteristics and socio-economic factors, which are not shown here. The second piece of information concerns the health data, some of which is accessible to UK Biobank researchers through a standard application. For instance, the main database includes inpatient records of the first occurrences of a series of clinical adverse events. An example with pseudo-data is provided in Table 1b, including the same pseudo-IDs of the subject, as well as the ICD-10 codes and dates of the events.

**Table 1:** Example of pseudo cohort data, including **a** baseline cohort information, **b** health outcomes, and **c** residential histories.

(a) Cohort info		
Subject ID	Enrolment date	Last follow-up date
1	May 1, 2007	March 12, 2017
2	April, 14, 2009	September 25, 2019
3	November 23, 2006	Present

(b) Inpatient visit outcomes table by subject		
Subject ID	ICD	Date
1	E11	April 23, 2012
1	I20	July 4, 2013
1	I21	September 30, 2016
2	C34	February 24, 2010
3	J40	March 14, 2007
3	J41	April 11, 2008
3	J43	May 22, 2009

(c) Residential histories					
Subject ID	Location ID	Start date	End date	Easting	Northing
1	Loc_12	April 1, 2005	May 22, 2012	515,200	184,800
1	Loc_43	May 23, 2012	March 12, 2017	384,800	394,100
2	Loc_92	December 18, 2007	September 3, 2009	342,700	387,100
2	Loc_6	September 4, 2009	April 3, 2017	528,100	105,600
2	Loc_24	April 4, 2017	September 25, 2019	459,900	450,700
3	Loc_87	November 20, 1994	Present	177,500	314,500

The final piece of information is the residential histories of the subjects. In the UK Biobank, these are limited-access data, represented by the dates and locations of the participants’ residential addresses, where the location represents the centroid of a 1 km and 100 m buffer that contains the exact location. These data were collected during the baseline interview and are ongoingly updated via self-report or new registration to general practices of the National Health Service (NHS). Residential pseudo-data are shown in Table 1c, including pseudo-IDs for subjects and locations, and start/end dates of the period the subject stayed at each address, alongside the corresponding geographical coordinates (in Northing-Eastings values of the British National Grid).

### Spatio-temporal exposure maps

Advances in exposure assessment have been achieved through important developments in two areas. First, the increasing availability of data resources with high spatial and temporal resolution and extended coverage, in particular from remote sensing sources. Second, the provision of innovative analytical techniques, for instance, machine learning algorithms or atmospheric and climate models with increasingly better performance and reliability. These technological advancements make it possible to produce fine-scale spatio-temporal maps of environmental exposures applicable in population-based epidemiological studies [15]. These state-of-the-art tools have rapidly substituted classical exposure assessment methods, such as the assignment to the closest monitoring station or traditional land-use regression models, as the latter fail to provide accurate estimates for large areas and over long periods of time [16].

In this contribution, we consider a dataset that is currently used to assign daily exposures to fine particulate matter (PM_2.5_, in µg/m^3^) to the participants locations of the UK Biobank. This product was generated by a multi-stage machine learning model that was applied to predict daily PM_2.5_ concentrations in a 1 × 1 km grid across Great Britain during the period 2008–2018. The model was trained using data from 581 monitoring stations, using a long list of spatial and spatio-temporal predictors including remote sensing satellite observations, traffic data, weather simulations, road characteristics, and land-use information, among others. The model had a good overall performance, with a cross-validated *R*^2^ of 0.767. Details are provided elsewhere [16].

This resource is used in the next sections to exemplify the linkage process of PM_2.5_ measures to participants of the UK Biobank.

### Spatial linkage (Step 1)

Geographical information systems (GIS) have become a staple technique for constructing environmental databases. In this context, GIS provide a binding framework between environmental measures and cohort data collected at the individual level, combining different layers of information to a single point in space [17]. These techniques are employed in epidemiological analyses by overlying geographical reference grids over which the investigators can jointly map exposure information with individual or area-level variables. This allows maximising the available information by downscaling or upscaling measurements across levels of aggregation, as well as combining measurements across space and time.

**Fig. 1:**
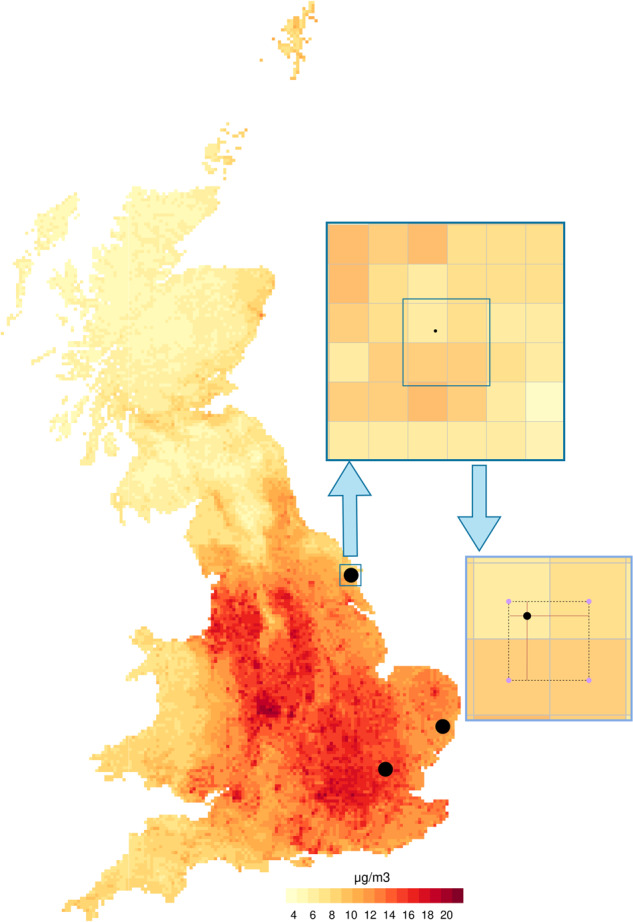
The maps display PM_2.5_ levels on a specific day over Great Britain, with three locations (large black dots) that represent the residential addresses of a specific subject (ID 2 in Table 1). The magnified area on top represents the exact location at higher resolution, surrounded by the four nearest centroids (small indigo dots) of the overlaid PM_2.5_ grid. Without interpolation, the residential exposure value (small black dot) would be represented by the value of the nearest centroid. The magnified area below illustrates the process of reconstructing the residential value as a bilinear interpolation of the four nearest centroids.

We discuss the application of GIS techniques and related problems by illustrating the linkage of environmental exposures to the UK Biobank. The cohort database includes the locations of the residential addresses of each participant. An example is provided in Fig. 1, which shows the PM_2.5_ levels for one day from the 1 × 1 km gridded spatio-temporal map presented in the previous section. The map also includes the three residential addresses for Subject 1 listed in Table 1b, and for one address, it adds a magnified detail of the 1 × 1 km cells surrounding the location.

A simple linkage option is to assign the value of the grid cell containing the location. However, this option has two main drawbacks. First, it does not account for the information of the neighbouring cells, which can complement the cell-level measurement with details on the small-scale variability and improve the exposure assignment. Second, and more importantly, the direct linkage of cell-specific values can result in potential privacy breaches described above by allowing back-tracing of the location using geographic information from the original gridded environmental data, if this is publicly available and at sufficiently high resolution.

In lieu of the simple linkage approach described above, other methods of varying complexity can be used and the choice depends on the type of exposure data and the underlying objective of data linkage. For example, in the presence of ground monitor data, a simple strategy would be to assign exposure as the inverse-distance weighted average of the nearby monitors. For gridded exposure data, established routines such as simple spatial averaging, bilinear and kriging interpolation exist in the two-dimensional case, while more specific methods have been investigated more recently as a consequence of the raise of new forms of spatial data [18]. Here, we propose the use of the bilinear interpolation, which consists of a repeated linear interpolation across the two geographical dimensions and it is graphically represented in Fig. 1. We deem this method to be an effective but simple option, among the others, for several reasons. The process addresses the two drawbacks of the simpler linkage described above: first, it preserves the exposure information by spatially combining measurements across multiple grid cells. Second, and more importantly, it generates a continuous exposure field with values that cannot be linked back to the original sources, preventing the identification of the residential locations even when using highly resolved and public exposure databases. Compared to other interpolation methods, bilinear interpolation does not require a choice of the parameters (e.g., search radius or number of neighbours) and it is more accurate than simple spatial averaging as it accounts for the distances among the points in the computation of the interpolated value [19]. Moreover, its deterministic nature makes it computationally inexpensive even for very large datasets, for instance in comparison to kriging [20]. Finally, bilinear interpolation is commonly implemented in data analysis and geographical software and therefore easy to apply. It must be highlighted that, regardless of the method, the accuracy of this linkage would depend on the spatial resolution of the original exposure data, and the precision of the coordinates for the locations.

### Reconstruction of individual-level exposure series (Step 2)

The linkage-interpolation operation in the previous section can be performed for each residential location of each participant of the cohort. The output data, combined with the residential histories, allow reconstructing subject-specific series representing individual exposure profiles.

**Fig. 2:**
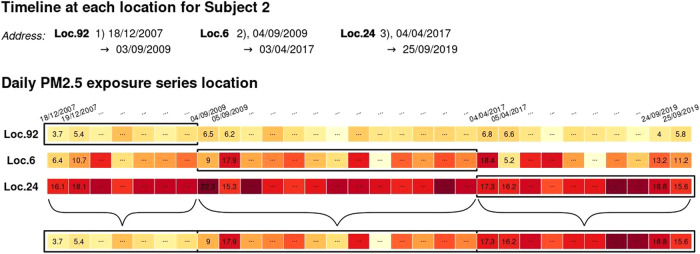
The top three series represent the sequences of daily exposures at the residential addresses of subject ID 2. At the bottom, the final subject-specific exposure series is assembled by concatenating the three series above based on the respective residential periods.

This step is illustrated in Fig. 2 for Subject 2 in our case study. Specifically, the residential histories of this subject reported in Table 1c, combined with the interpolated series for the three residential locations obtained following the procedure in Fig. 1, allow extracting blocks of exposure series corresponding to the timeline of each subject’s residence at specific addresses. These blocks are then merged into a single individual series that represents a detailed residential exposure profile for an individual, accounting for exposure levels experienced at different locations during a defined time interval.

### Definition of individual summaries for epidemiological studies (Step 3)

The reconstruction of exposure profiles in the previous section offers detailed individual-level time series characterised by a fine temporal disaggregation, allowing the definition of various exposure summaries. In epidemiological analyses, this is of particular relevance as such summaries can be flexibly tailored to the specific research questions and study designs, resulting in more informative inferential procedures and reducing exposure misclassification.

The definition of the exposure summaries first requires assumptions on the temporal dependency between exposure and outcomes, determined by underlying biological mechanisms. Two intertwined aspects are particularly relevant: the timescale of the association and the related exposure window. The former differentiates short-term risks associated with daily variation from long-term effects due to chronic exposures experienced over years or decades. The latter determines the maximal temporal interval over which the exposure exerts its action, within a specific timescale.

**Fig. 3:**
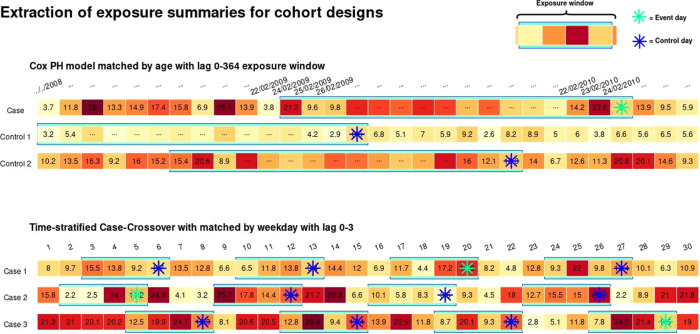
The graph presents the use of the exposure data in two examples of study designs used in environmental epidemiology. The top figure illustrates a risk set within a study on the incidence of lung cancer (ICD-10: C34) with a case (subject 2) and controls matched by age used in a Cox proportional hazard model to estimate long-term risks. The event (aquamarine star) and control (blue star) times are used to reconstruct backwards the exposure profiles in the three subjects, defined as 365-day (lag 0–364) averages of PM_2.5_ (light blue boxes). The bottom figure displays the same process to define risk sets for a time-stratified case-crossover to estimate short-term risks. The graph shows three separate subjects (unrelated to Table 1) with the event (aquamarine star) and controls (blue star) days matched on the day of the week in the same month, with exposure profiles defined as averages of lag 0–3.

We use our case study to illustrate the definition of exposure summaries for two different study designs for individual-level data: a survival analysis based on Cox proportional hazard models to assess long-term effects [21], and a case-crossover analysis to investigate short-term associations [22]. The two examples are represented in Fig. 3, using the pseudo-data related to specific health events in Table 1b.

The Cox proportional hazard model is based on a between-subject comparison, defining separate risk sets for each event. Each risk set includes the case subject as well as a series of control subjects who are at risk at the time of the event. An example of a single risk set is shown at the top of Fig. 3. The composition of the risk set depends on the time axis of interest, which in this case is represented by the age of the subjects. The controls are therefore sampled when they reach the same age that the case had when experiencing the event. For each subject, we retrieve their exposure history backwards with a lag period equal to the exposure window, and therefore define the related exposure summary.

A case-crossover design follows a similar extraction procedure. However, in contrast to the survival model above, the latter is based on a within-subject comparison, and the case and controls are represented by different times within the follow-up period of the same subject. Several control sampling schemes have been proposed in the literature [23] with the most common being the time-stratified scheme with controls sampled within pre-specified strata. An example with three subjects representing three separate risk sets with an exposure window of four days (lag 0–3) is provided at the bottom of Fig. 3.

The availability of finely stratified temporal profiles allows higher precision in the definition of the exposure windows, before any potential aggregations are performed. For instance, multiple lag terms can be defined using daily, monthly, or yearly strata, thus allowing the application of distributed lag models over different timescales [24].

## Discussion

This article describes a framework to process and link environmental exposures to cohort studies. The methodology can be applied to retrieve detailed individual-level exposure profiles, hence allowing the application of flexible epidemiological study designs to investigate health risks associated with environmental stressors. The paper conceptualises several steps and methodological aspects, with illustration in a case study featuring the UK Biobank cohort using simplified pseudo-datasets. The framework has broad applications and can be used to complement cohort databases with high-resolution spatio-temporal exposure measurements, enabling to investigate complex aetiological questions between environmental factors and health.

This work can contribute to clarify and improve on current limitations in the research field. An example is offered by recent cohort analyses of associations between low levels of air pollution with mortality and morbidity conducted in the USA, Canada, and Europe [6]. These investigations applied state-of-the-art methodologies to large population-based cohort databases, representing milestones in air pollution epidemiology. Specifically, the North American studies examined health risks associated with several air pollutants by reconstructing exposures with resolved spatial predictions and various temporal disaggregation [11]. However, these cohort analyses often relied on administratively collected cohort data whereby, due to privacy constraints, exposure information could only be matched to large administrative areas. In contrast, recent multi-cohort European studies [13] took advantage of exposure models with high spatial resolution and linkage at residential level. However, the exposure data was not temporally disaggregated, and the analyses relied on simple exposure summaries based on averages for specific numbers of years, preventing the investigation of complex temporal dependencies. The framework presented here, given the availability of the data, helps addressing these limitations, providing a privacy-protecting approach to safely link resolved spatio-temporal exposure maps to large databases with rich individual information, thereby improving the design of cohort studies.

The example based on the UK Biobank cohort also highlights some practical problems. First, our choice of the interpolating method was based on practical criteria, but in general this decision would benefit from rigorous comparisons, for instance based on statistical goodness of fit measures [19]. Second, the linkage procedure exemplified necessitates information on residential mobility. Currently, in the UK Biobank such data is only reconstructed from participants’ self-reports and NHS contacts. This process is error-prone and can entail exposure misclassification. Third, the accuracy of the exposure assessment depends on the quality and resolution of the spatio-temporal exposure models. In our example, we demonstrated a linkage with gridded databases of pollution derived from moderate-to-high predictive performance, which similarly provides an imperfect characterisation of exposure levels. Finally, even when accurately representing residential levels, outdoor estimates are only a proxy of the actual personal exposures.

Nonetheless, the framework described here offers a template for future developments to address current limitations and overcome new challenges. Most importantly the approach can be extended beyond the linkage of residential measurements, for instance incorporating activity-based models or personal monitoring campaigns to improve individual exposure assessment in different environments [25]. This is relevant as hyperlocal exposure models are increasingly deployed in urban settings with the aim of addressing environmental disparities [26] and the environmental datasets can be made publicly available to researchers [27]. Finally, the assignment of individual-level exposure profiles can be replicated for multiple stressors. This will allow the investigation of health risks associated with the bulk of environmental exposures, consistent with the notion and research paradigm of the exposome [28]. In this context, the linkage framework we illustrated can be applied and further developed to finely reconstruct detailed exposure information across large cohorts and long study periods, while at the same time preventing confidentiality breaches by providing bespoke exposure levels that cannot be traced back to the original data.

## Data Availability

The code and synthetic data for reproducing a simpler version of the illustrative example is made available by the authors in a GitHub repo (https://github.com/gasparrini/EnvExpLink). The analysis was performed in the R software environment.
