# Correction: Reconstructing individual-level exposures in cohort analyses of environmental risks: an example with the UK Biobank

**DOI:** 10.1038/s41370-025-00771-5

**Published:** 2025-04-16

**Authors:** Jacopo Vanoli, Malcolm N. Mistry, Arturo De La Cruz Libardi, Pierre Masselot, Rochelle Schneider, Chris Fook Sheng Ng, Lina Madaniyazi, Antonio Gasparrini

**Affiliations:** 1 School of Tropical Medicine and Global Health, Nagasaki University, Nagasaki, Japan; 2 Environment & Health Modelling (EHM) Lab, Department of Public Health Environments and Society, London School of Hygiene & Tropical Medicine, London, UK; 3 Department of Economics, Ca’ Foscari University of Venice, Venice, Italy; 4 Φ-lab, European Space Agency, Frascati, Italy; 5 Department of Global Health Policy, Graduate School of Medicine, The University of Tokyo, Tokyo, Japan

Correction to: *Journal of Exposure Science & Environmental Epidemiology* 10.1038/s41370-023-00635-w, published online 8 January 2024

**Data availability**

The code and synthetic data for reproducing a simpler version of the illustrative example is made available by the authors in a GitHub repo (https://github.com/gasparrini/EnvExpLink). The analysis was performed in the R software environment.

The original article has been updated.

